# Supplementary material for: Structure‐energy‐based predictions and network modelling of RASopathy and cancer missense mutations
Source: Mol Syst Biol. 2014 May 6;10(5):727. doi: 10.1002/msb.20145092 (PMC4188041; doi:10.1002/msb.20145092)
Supplement: Supplementary file 1 — Supplementary Figure S1 [file MSB-10-5-727-s1.pdf]

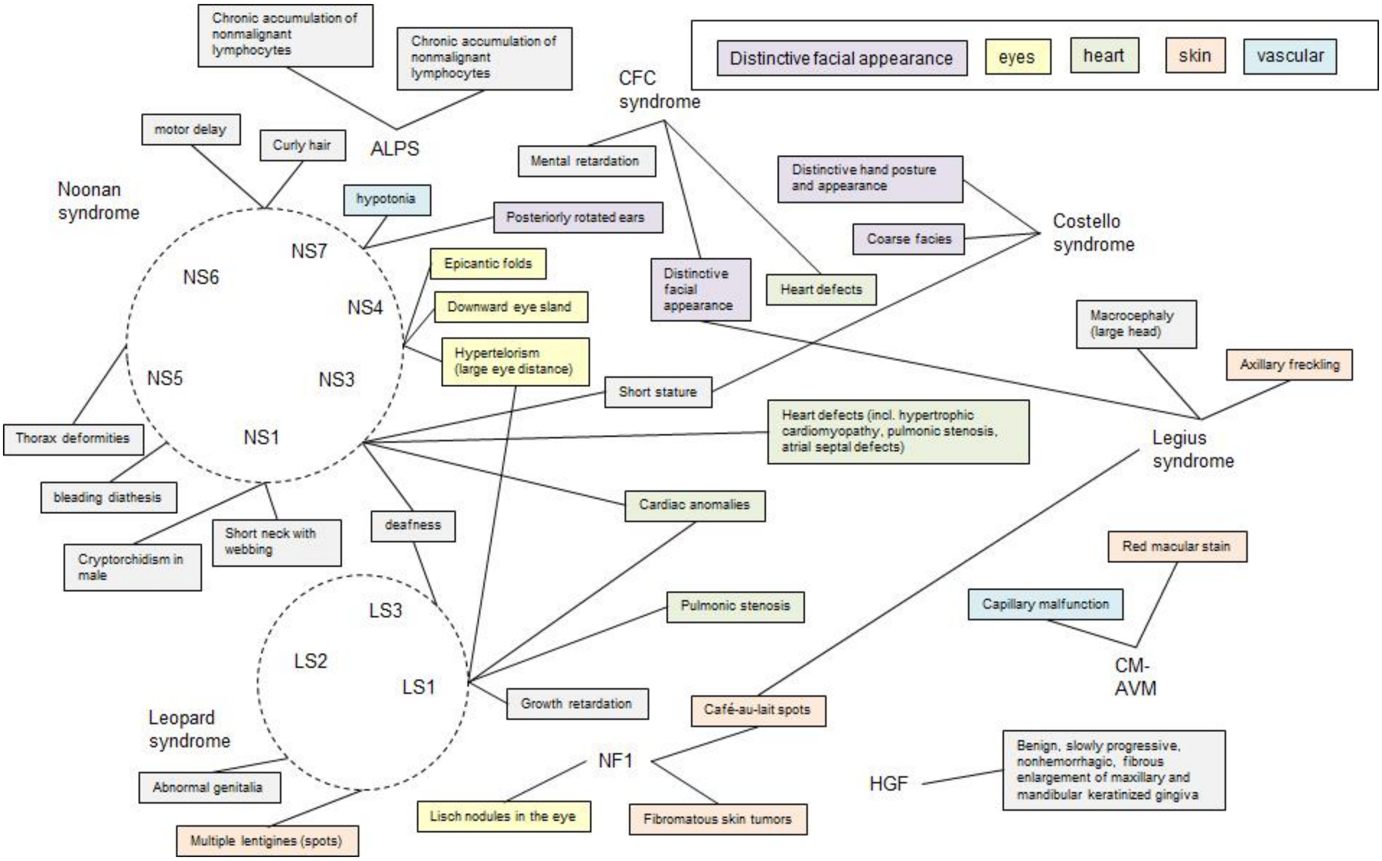

Supplementary Figure S1. Disease symptoms of RASopathies. The information on disease symptoms was extracted from the OMIM database. Symptoms were roughly classified according to the region in the body that is affected. Abbreviations: NS, Noonan syndrome; NF1, neurofibromatosis type 1; CFC, cardiofaciocutaneous; LS, LEOPARD syndrome; HGF, hereditary gingvial fibromatosis; CM-AVM, capillary malfunction - arteriovenous malfunction; ALPS, autoimmune lymphoproliferative syndrome.
