# Supplementary material for: Structure‐energy‐based predictions and network modelling of RASopathy and cancer missense mutations
Source: Mol Syst Biol. 2014 May 6;10(5):727. doi: 10.1002/msb.20145092 (PMC4188041; doi:10.1002/msb.20145092)
Supplement: Supplementary file 2 — Supplementary Figure S2 [file MSB-10-5-727-s2.pdf]

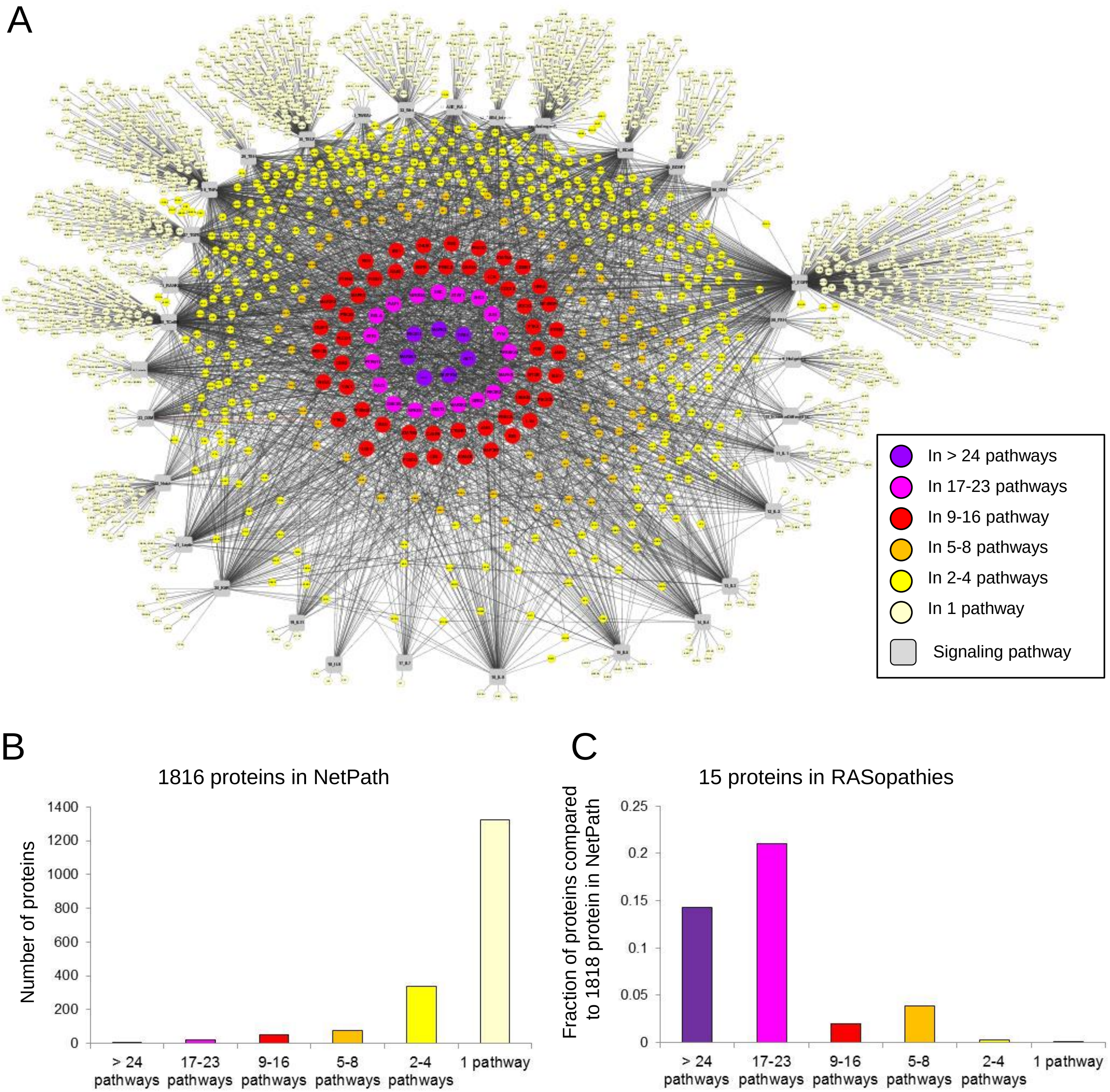

Supplementary Figure S2. Participation of 1816 proteins in 33 Netpath signaling pathways. (A) In the network (upper part), the colour and size of the nodes represents the number of signalling pathways they predicate in (see legend). (B) Graph showing the number of participating proteins in different pathways for all 1816 proteins in NetPath. (C) Graph showing the number of participating proteins (divided by the total number of proteins in each category from panel A) for the 15 proteins involved in RASopathies.
