# Supplementary figures and images for: Structure‐energy‐based predictions and network modelling of RASopathy and cancer missense mutations
Source: Mol Syst Biol. 2014 May 6;10(5):727. doi: 10.1002/msb.20145092 (PMC4188041; doi:10.1002/msb.20145092)

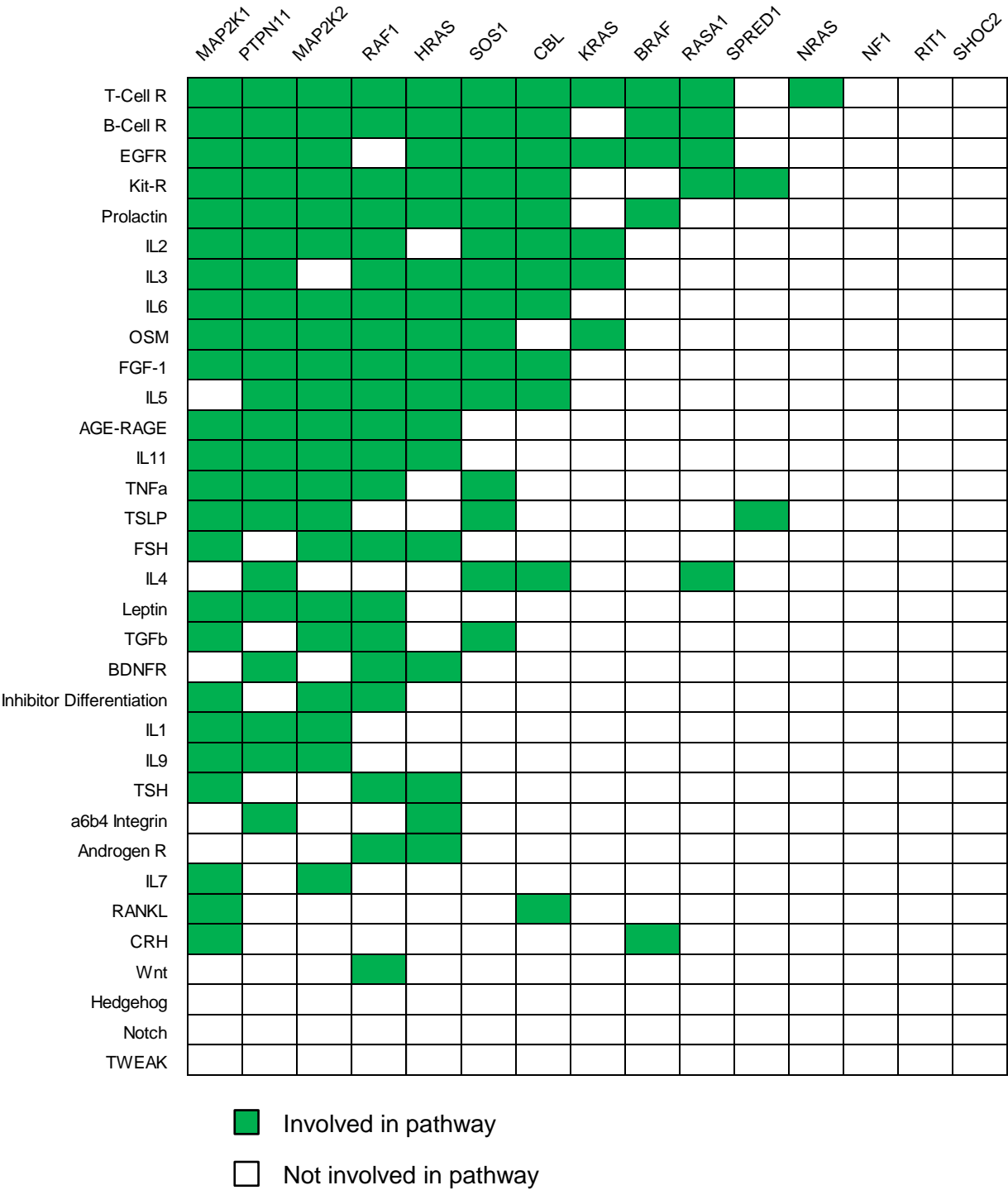

Supplement: Supplementary file 3 — Supplementary Figure S3 [file MSB-10-5-727-s3.pdf]
