# Supplementary material for: Structure‐energy‐based predictions and network modelling of RASopathy and cancer missense mutations
Source: Mol Syst Biol. 2014 May 6;10(5):727. doi: 10.1002/msb.20145092 (PMC4188041; doi:10.1002/msb.20145092)
Supplement: Supplementary file 4 — Supplementary Figure S4 [file MSB-10-5-727-s4.pdf]

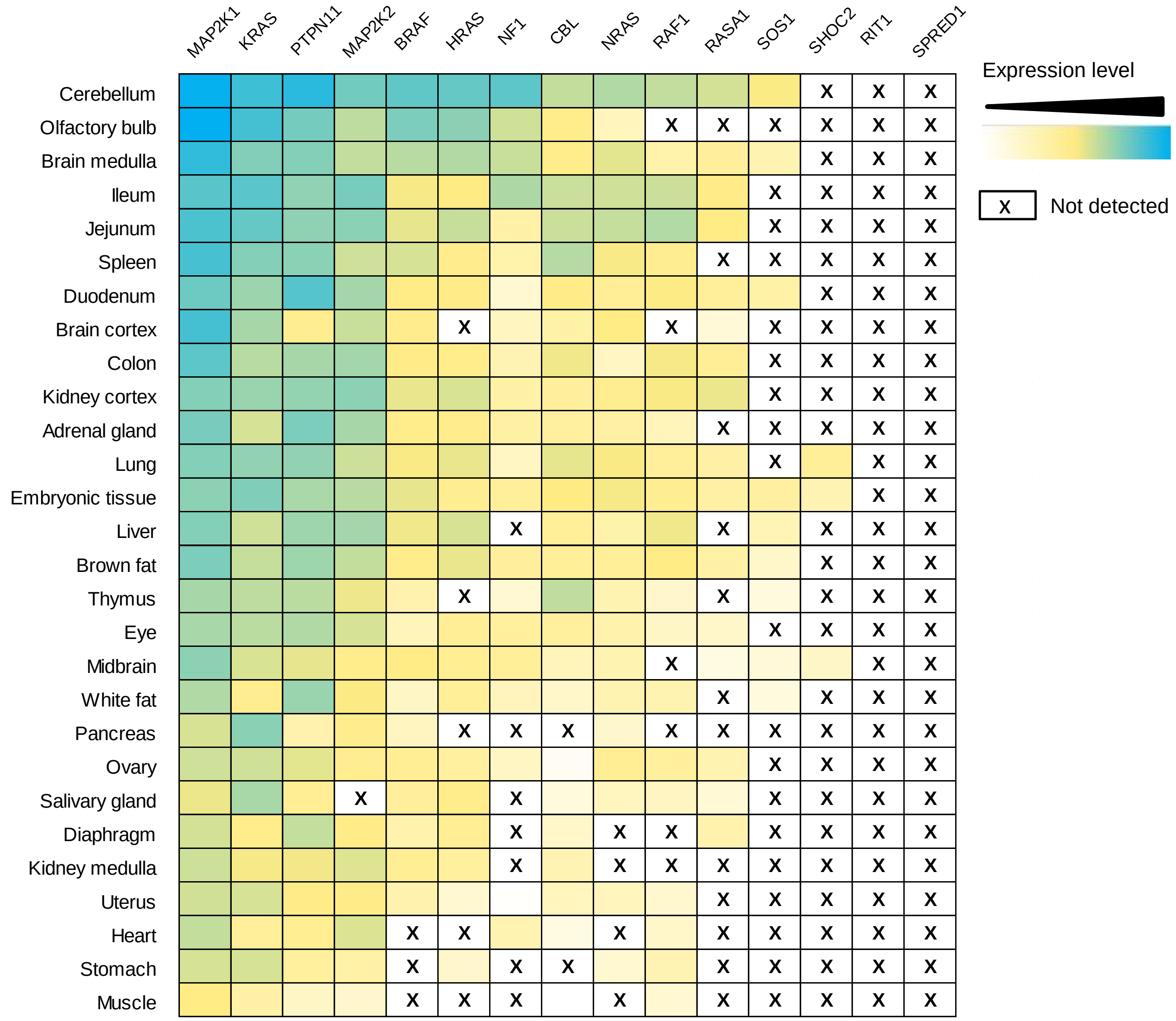

Supplementary Figure S4. Protein expression levels in 28 tissues for the 15 RASopathy genes. Expression levels (based on Geiger et al, Mol Cell Proteomics, Jun;12(6):1709-22, 2013) are colored from yellow to blue with increasing value. Black crosses indicate no detection of the respective protein.
