# Supplementary material for: Structure‐energy‐based predictions and network modelling of RASopathy and cancer missense mutations
Source: Mol Syst Biol. 2014 May 6;10(5):727. doi: 10.1002/msb.20145092 (PMC4188041; doi:10.1002/msb.20145092)
Supplement: Supplementary file 5 — Supplementary Figure S5 [file MSB-10-5-727-s5.pdf]

	MAP2K1	RASA1	RAF1	PTPN11	KRAS	SHOC2	HRAS	SOS1	RIT1	NF1	NRAS	CBL	MAP2K2	BRAF	SPRED1
Superior cervical ganglion	133	783	1430	832	1622	1165	304	580	765	743	726	667	206	X	X
Amygdala	2870	1534	674	1521	1097	665	595	337	199	122	154	87	59	X	X
Prefrontal cortex	1262	2057	1025	1602	1027	863	552	215	191	220	164	177	50	X	X
Occipital lobe	2377	1291	867	1662	1093	751	399	216	220	130	244	92	53	X	X
Subthalamic nucleus	2098	1300	728	1426	961	690	666	254	387	80	170	360	90	X	X
Parietal lobe	1808	1216	667	1662	867	777	518	336	383	108	297	141	120	X	X
Placenta	693	4922	931	434	448	259	162	223	213	129	169	81	43	X	X
Medulla oblongata	1606	1361	650	1439	624	662	698	292	212	279	291	340	64	X	X
Cingulate cortex	1661	1104	862	1164	897	704	492	221	338	304	203	265	64	X	X
Globuspallidus	1667	1169	570	981	942	661	491	306	259	549	87	299	113	X	X
Ciliary ganglion	829	890	1196	327	900	783	258	258	553	711	769	423	103	X	X
Whole brain	2623	956	416	1081	1040	374	780	165	139	77	120	83	43	X	X
Pons	1122	1192	635	1219	694	599	612	466	404	228	355	239	125	X	X
Caudatenucleus	1856	725	783	1636	392	443	689	277	277	83	258	136	121	X	X
Trigeminal ganglion	120	812	875	365	865	1054	843	282	362	727	820	281	137	X	X
Skeletal muscle	156	392	1303	542	791	667	639	505	596	641	139	526	238	X	X
Temporal lobe	1263	1046	595	996	762	680	567	83	289	164	363	213	102	X	X
DRG	390	934	832	499	1197	729	507	333	390	572	316	236	101	X	X
Atrioventricular node	122	745	860	423	879	680	374	328	595	739	291	443	92	X	X
Cerebellum	1870	536	1020	577	877	366	416	143	224	156	128	157	33	X	X
Cerebellum peduncles	1680	580	792	886	852	393	353	147	299	122	209	109	57	X	X
Hypothalamus	1262	968	572	1310	400	625	458	227	177	101	149	94	36	X	X
Thalamus	1501	718	718	1190	175	569	563	242	166	74	181	81	51	X	X
Testis leydig cell	304	1294	660	246	618	798	191	488	329	395	159	328	166	X	X
Testis interstitial	329	1301	793	303	418	686	235	430	294	228	161	577	51	X	X
Testis germ cell	330	1284	825	429	379	927	194	399	229	225	165	325	46	X	X
Testis seminiferous tubule	388	1089	815	260	571	984	62	355	252	258	129	278	69	X	X
Fetal brain	561	1032	918	643	484	594	370	220	109	126	126	101	45	X	X
Spinalcord	494	748	635	1326	242	393	306	202	236	81	113	120	43	X	X
Olfactory bulb	517	635	599	494	876	400	323	191	248	220	125	149	27	X	X
Fetal liver	430	537	751	467	575	350	108	156	191	128	290	143	77	X	X
Skin	179	564	772	269	452	419	217	331	230	362	153	145	57	X	X
Thyroid	315	728	645	480	313	414	241	232	153	94	104	243	49	X	X
Whole blood	490	412	1176	249	345	771	67	107	193	30	63	50	17	X	X
Appendix	265	450	236	414	374	557	255	365	217	315	286	179	49	X	X
Colorectal adenocarcinoma	372	321	511	588	705	276	417	196	137	85	113	176	34	X	X
Uterus	214	511	555	804	496	433	183	179	148	213	82	64	32	X	X
721_B_lymphoblasts	908	412	423	580	471	349	196	94	94	51	183	38	10	X	X
Kidney	507	272	849	289	394	260	196	153	290	177	191	88	62	X	X
BM-CD33+Myeloid	646	354	622	317	607	474	175	151	114	22	135	65	10	X	X
Trachea	464	474	463	362	427	325	176	182	195	251	161	130	51	X	X
Testis	352	468	550	103	440	573	138	195	253	80	137	261	50	X	X
Adipocyte	428	352	411	711	310	355	163	419	128	55	147	62	47	X	X
Cardiac myocytes	539	263	536	319	389	394	495	85	137	104	202	60	34	X	X
Pituitary	366	658	270	518	303	574	135	183	149	180	73	51	39	X	X
Uterus corpus	322	386	539	278	286	327	241	237	287	182	229	96	58	X	X
Prostate	541	326	454	302	645	218	230	170	131	161	119	86	42	X	X
Tongue	296	390	496	293	365	397	414	136	205	166	35	129	41	X	X
BM-CD71+EarlyErythroid	383	238	585	429	422	421	170	95	132	67	156	75	30	X	X
PB-CD56+NKCells	434	237	352	472	672	428	103	167	74	39	122	25	43	X	X
Fetal lung	410	500	629	442	301	232	143	111	170	46	89	68	26	X	X
Adrenal cortex	234	260	443	254	278	364	242	267	217	286	76	147	49	X	X
Fetal thyroid	225	339	781	262	362	283	263	154	42	148	38	148	35	X	X
Bone marrow	540	182	695	127	356	263	54	92	232	62	132	163	50	X	X
Adrenal gland	447	289	518	337	219	203	174	130	134	140	126	170	30	X	X
PB-BDCA4+Dentritic_Cells	837	218	334	313	281	332	90	148	56	15	162	82	9	X	X
Bronchial epithelial cells	585	330	261	389	175	193	325	83	146	58	162	60	13	X	X
Tonsil	423	380	333	193	249	309	165	188	148	120	107	89	44	X	X
Thymus	376	197	516	192	457	192	120	119	137	56	125	79	19	X	X
Liver	570	115	447	119	179	141	171	112	163	174	104	235	50	X	X
Pancreatic Islets	442	275	295	270	275	334	145	158	100	81	63	58	31	X	X
Lympho maburkitts raji	437	259	236	398	333	210	250	111	36	81	60	56	38	X	X
Leukemia lymphoblastic (molt4)	413	220	495	317	322	174	101	65	87	45	118	66	21	X	X
Salivary gland	239	454	312	266	185	286	120	130	125	94	92	86	41	X	X
Lymphnode	369	466	410	192	159	235	119	138	95	51	84	83	17	X	X
Smooth muscle	437	333	214	456	190	285	106	52	102	31	117	46	14	X	X
Lung	527	121	497	160	313	126	167	128	129	59	65	57	30	X	X
PB-CD8+Tcells	399	237	369	268	366	218	77	232	51	20	88	30	9	X	X
PB-CD4+Tcells	373	271	357	212	334	279	89	203	36	60	53	50	8	X	X
Heart	259	129	681	47	247	169	323	78	102	105	45	58	64	X	X
PB-CD14+monocytes	493	180	446	149	385	281	76	68	96	14	58	26	16	X	X
BM-CD105+endothelial	129	260	357	499	404	199	134	79	33	13	88	16	32	X	X
Pancreas	246	257	289	214	285	254	151	111	123	104	71	92	27	X	X
BM-CD34+	126	218	397	487	383	241	66	141	39	15	64	25	8	X	X
leukemia promyelocytic(hl60)	463	223	261	297	196	167	80	72	40	45	79	39	20	X	X
Ovary	218	271	187	153	146	197	59	49	58	122	75	86	25	X	X
lymphoma burkitts daudi	283	185	266	192	100	185	93	81	88	30	58	43	35	X	X
leukemia chronicmyelogenous (k562)	221	211	189	254	169	67	19	121	82	58	104	26	26	X	X
PB-CD19+Bcells	252	143	212	127	184	335	81	76	26	21	45	8	25	X	X

Expression level

X Not detected

Supplementary Figure S5. Transcript expression levels in 79 tissues for the 15 RASopathy genes. Expression levels (based on Su et al, Proc Natl Acad Sci U S A. Apr 2;99(7):4465-702002) are colored from yellow to blue with increasing value. Black crosses indicate no detection of the transcript.
