# Supplementary material for: Structure‐energy‐based predictions and network modelling of RASopathy and cancer missense mutations
Source: Mol Syst Biol. 2014 May 6;10(5):727. doi: 10.1002/msb.20145092 (PMC4188041; doi:10.1002/msb.20145092)
Supplement: Supplementary file 6 — Supplementary Figure S6 [file MSB-10-5-727-s6.pdf]

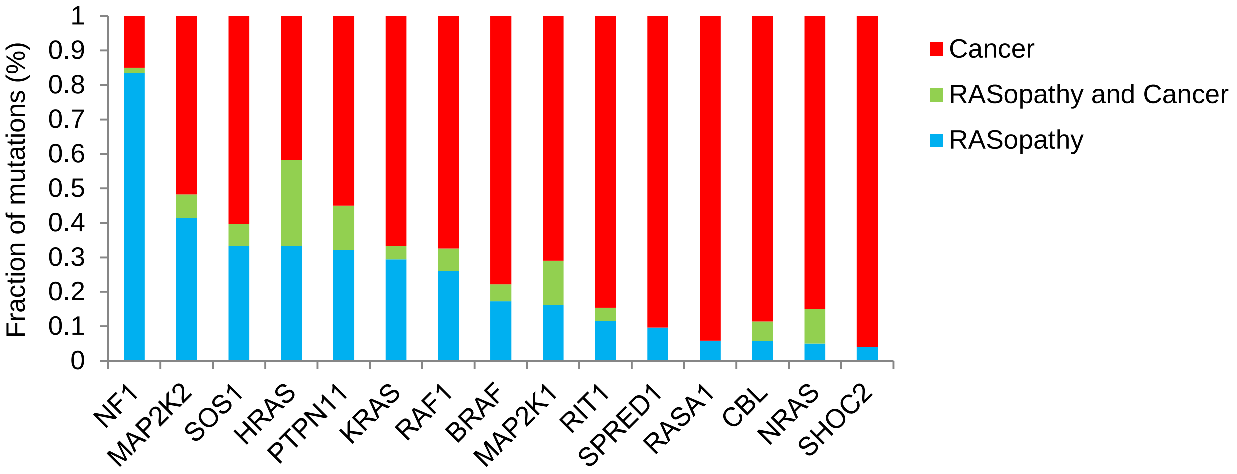

Supplementary Figure S6. Fraction of different RASopathy and cancer mutations in each of the 15 genes.
