# Supplementary material for: Structure‐energy‐based predictions and network modelling of RASopathy and cancer missense mutations
Source: Mol Syst Biol. 2014 May 6;10(5):727. doi: 10.1002/msb.20145092 (PMC4188041; doi:10.1002/msb.20145092)
Supplement: Supplementary file 7 — Supplementary Figure S7 [file MSB-10-5-727-s7.pdf]

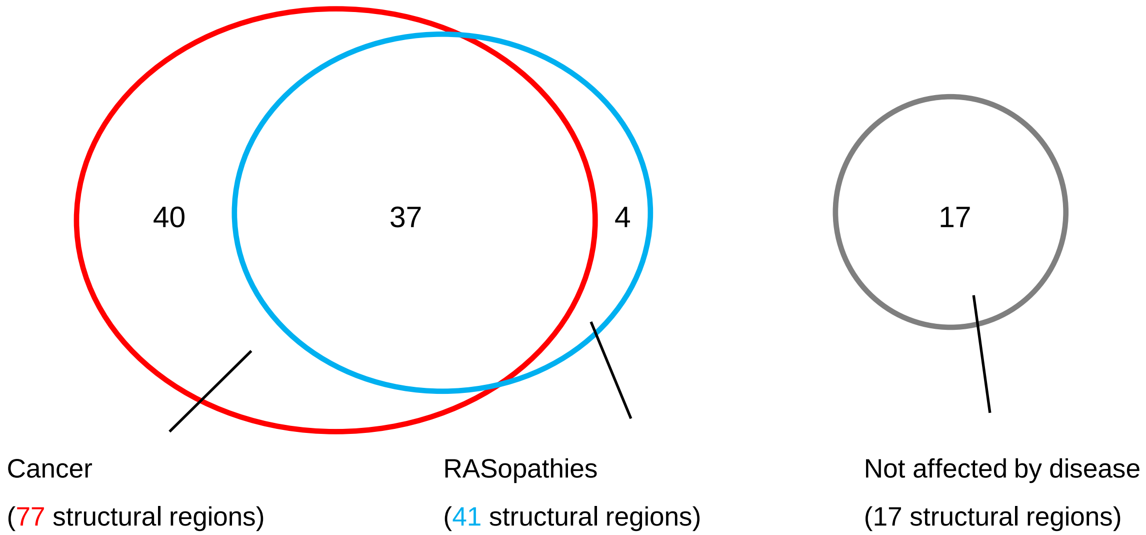

Supplementary Figure S7. Summary of the distribution of somatic and germline mutations in 98 different structural domains and inter-structural regions. Summary of the co-occurance of germline and RASopathy mutations in different structural regions as shown in Figure 2.
