# Supplementary material for: Structure‐energy‐based predictions and network modelling of RASopathy and cancer missense mutations
Source: Mol Syst Biol. 2014 May 6;10(5):727. doi: 10.1002/msb.20145092 (PMC4188041; doi:10.1002/msb.20145092)
Supplement: Supplementary file 8 — Supplementary Figure S8 [file MSB-10-5-727-s8.pdf]

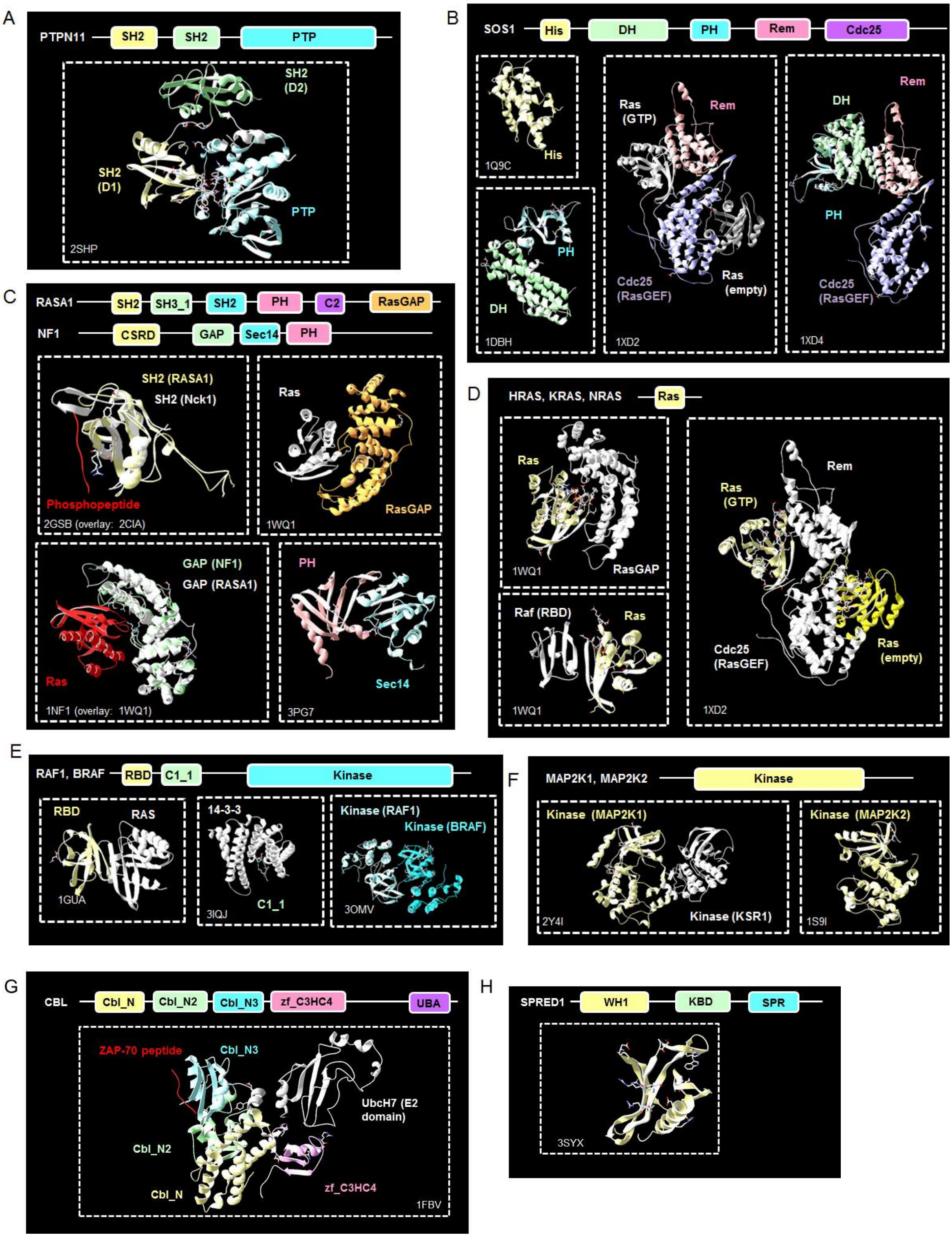


**Supplementary Figure S8.** Domain representation and structural coverage for the RASopathy-associated proteins. (**A**) Available 3D structures for PTPN11. (**B**) Available 3D structures for SOS1. (**C**) Available 3D structures for RASA1 and NF1. (**D**) Available 3D structures for HRAS, KRAS, and NRAS. (**E**) Available 3D structures for RAF1 and BRAF. (**F**) Available 3D structures for MAPK1 and MAPK2. (**G**) Available 3D structure for CBL. (**H**) Available 3D structure for SPRED1.
