# Supplementary material for: Structure‐energy‐based predictions and network modelling of RASopathy and cancer missense mutations
Source: Mol Syst Biol. 2014 May 6;10(5):727. doi: 10.1002/msb.20145092 (PMC4188041; doi:10.1002/msb.20145092)
Supplement: Supplementary file 9 — Supplementary Figure S9 [file MSB-10-5-727-s9.pdf]

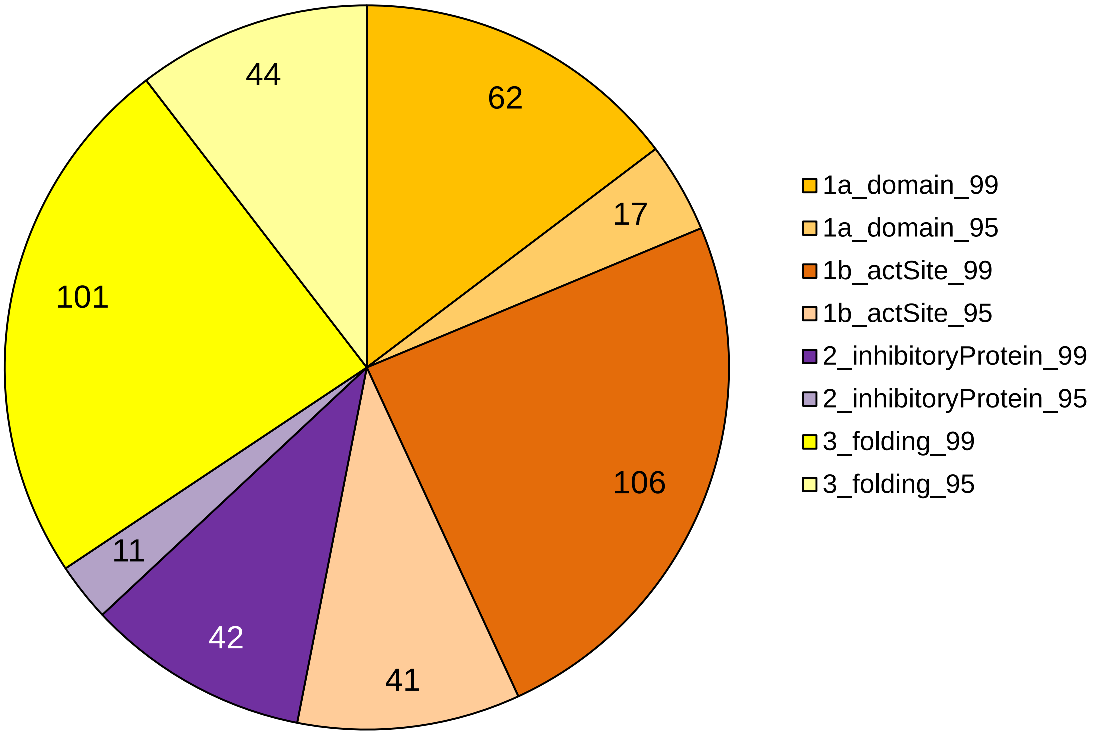

Supplementary Figure S9. Distribution of 424 missense mutations that map to a 3D structure into different classes with the two FoldX thresholds separately represented. The results of FoldX predictions are shown for 99% confidence predictions (2 STDEV =1.6 kcal/mol, and for 95% confidence predictions (1 STDEV = 0.8 kcal/mol). The different groups (1a_domain, 1b_actSite, 2_inhibitoryProtein, and 3_folding) follow the classification as shown in main figure 3. The label ‘99’ and ’95’ indicate the different confidence thresholds of FoldX energies.
