# Supplementary material for: Structure‐energy‐based predictions and network modelling of RASopathy and cancer missense mutations
Source: Mol Syst Biol. 2014 May 6;10(5):727. doi: 10.1002/msb.20145092 (PMC4188041; doi:10.1002/msb.20145092)
Supplement: Supplementary file 10 — Supplementary Figure S10 [file MSB-10-5-727-s10.pdf]

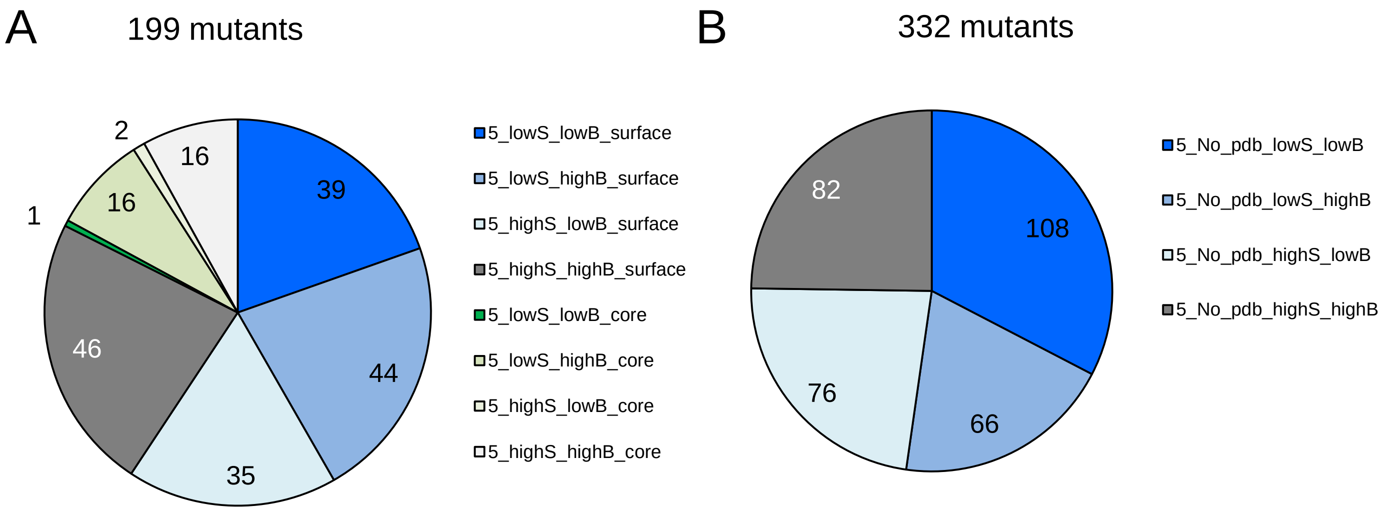

Supplementary Figure S10. Sequence-based classification for 199 non-destabilizing mutants and 332 mutations that could not be modeled on a three-dimensional structure. (**A**) Classification for non-destabilizing mutants. (**B**) Classification for mutations with no three-dimensional structural template. Abbreviation: S; Shannon entropy, B; Blosum matrix change, surface; mutations localized on the surface of the three-dimensional structure; mutations localized on the surface of the three-dimensional structure
