# Supplementary material for: Structure‐energy‐based predictions and network modelling of RASopathy and cancer missense mutations
Source: Mol Syst Biol. 2014 May 6;10(5):727. doi: 10.1002/msb.20145092 (PMC4188041; doi:10.1002/msb.20145092)
Supplement: Supplementary file 11 — Supplementary Figure S11 [file MSB-10-5-727-s11.pdf]

A

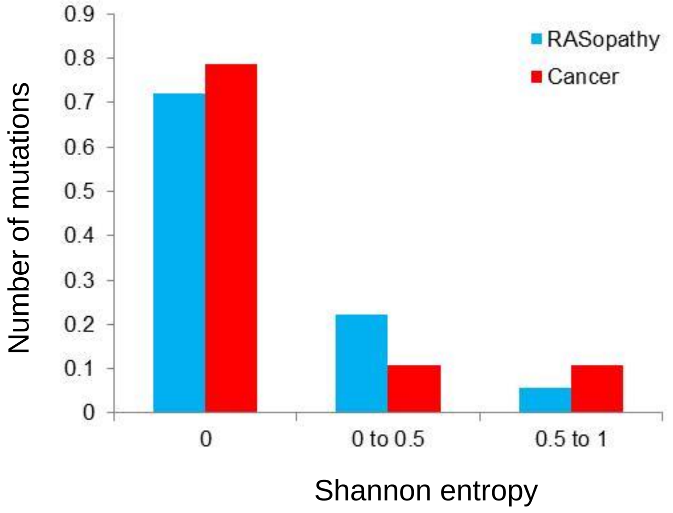

B

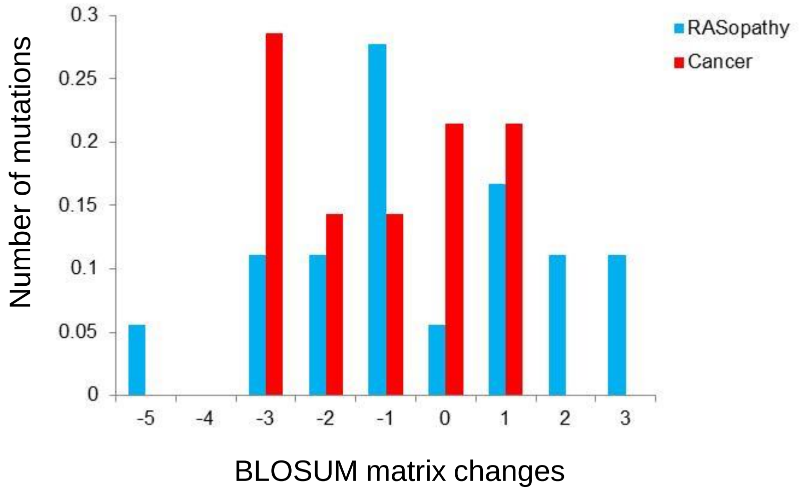

Supplementary Figure S11. Sequence-based analysis of a gold set of RASopathy and cancer missense mutations. (**A**) Shannon entropy values for amino acid positions. Higher values indicate less evolutionary conserved amino acid positions. (**B**) BLOSUM matrix changes for amino acid substitutions. Higher values indicate substitution with higher similar physiochemical properties of amino acids.
