# Supplementary material for: Structure‐energy‐based predictions and network modelling of RASopathy and cancer missense mutations
Source: Mol Syst Biol. 2014 May 6;10(5):727. doi: 10.1002/msb.20145092 (PMC4188041; doi:10.1002/msb.20145092)
Supplement: Supplementary file 12 — Supplementary Figure S12 [file MSB-10-5-727-s12.pdf]

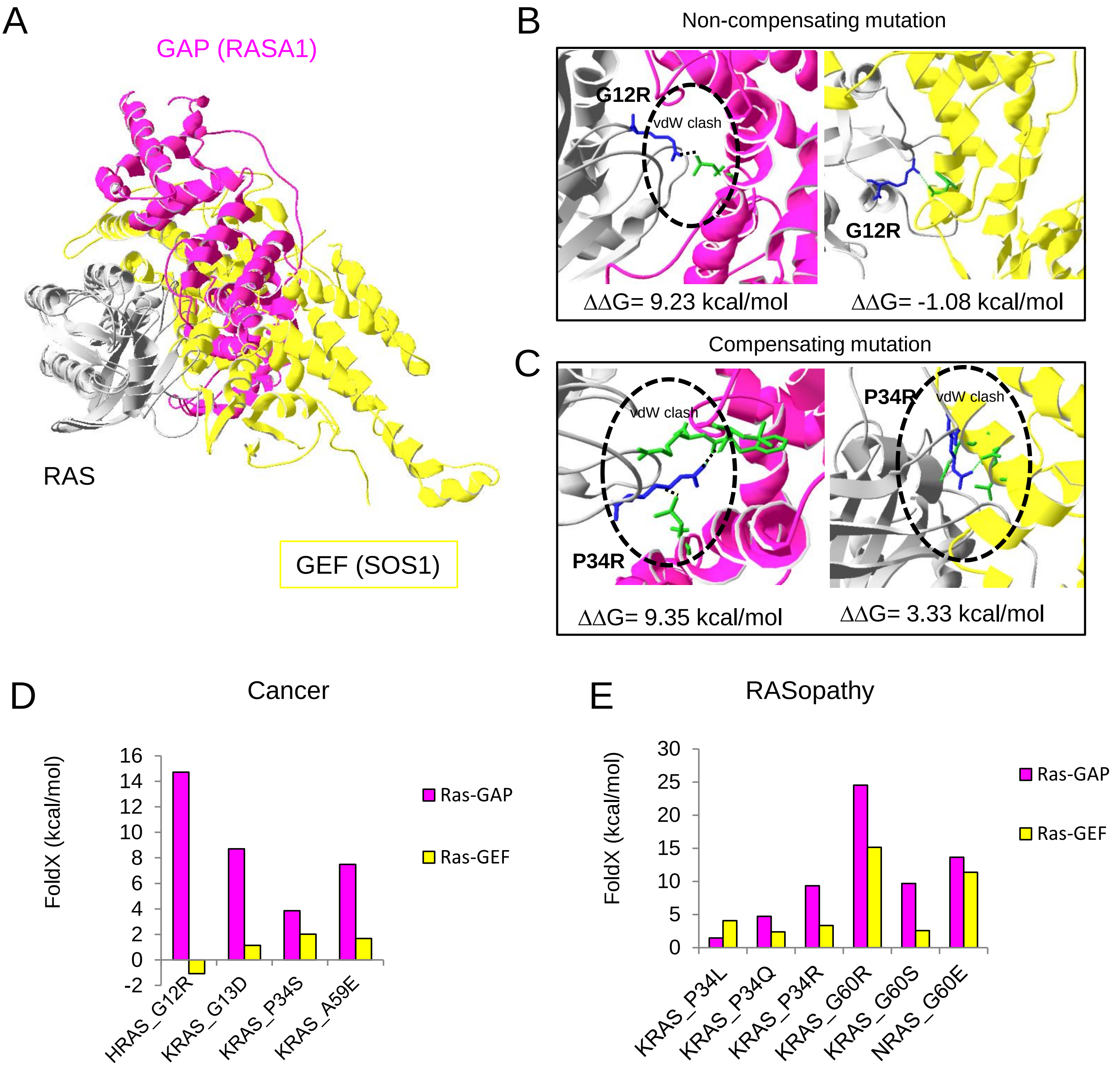

Supplementary Figure S12. FoldX-predicted compensatory mutations in Ras for binding to GEF and GAP. (**A**) Overlay of Ras in complex with a GEF (SOS1) and a GAP (RASA1). (**B**) Structural details for the RasG12R mutation as an example for a non-compensating mutation. Amino acid residues in Ras are shown in blue and neighbouring residues (<3 Å) of RASA1 and SOS1 in green. (**C**) Structural details for the RasP34R mutation as an example for a compensating mutation. Amino acid residues of Ras are shown in blue and neighbouring residues (<3 Å) in RASA1 and SOS1 in green. (**D**) FoldX energies for Ras cancer mutations modelled in complex with GAP (pdb entry: 1WGR) GEF (pdb entry: 1XD2, molecules B and C). (**E**) FoldX energies for Ras RASopathy mutations modelled in complex with GAP (pdb entry: 1WGR) GEF (pdb entry: 1XD2, molecules B and C).
