# Supplementary material for: Structure‐energy‐based predictions and network modelling of RASopathy and cancer missense mutations
Source: Mol Syst Biol. 2014 May 6;10(5):727. doi: 10.1002/msb.20145092 (PMC4188041; doi:10.1002/msb.20145092)
Supplement: Supplementary file 15 — Supplementary Table S3 [file MSB-10-5-727-s15.pdf]

Reaction Nr	Reaction	Rate [s-1] 1st order<br>Rate [M-1 s-1] 2nd order	Reverse rate [s-1]
1	RasD → RasT	Rate 1 (see table S4)	
2	RasT → RasD	Rate 2 (see table S4)	
3	RasD + GEF ↔ RasD_GEF	1.5e7	0.13
4	RasD_GEF→ GEF + RasT	Rate 4 (see table S4)	
5	RasT + GAP ↔ RasT_GAP	3e7	1
6	RasT_GAP → RasD + GAP	Rate 6 (see table S4)	
7	RasT + EFF ↔ RasT_EFF	2.9e6	Rate 7b (see table S4)

Supplementary Table S3. Reactions of the network model to simulate Ras WT and missense mutations.
