# Supplementary material for: Structure‐energy‐based predictions and network modelling of RASopathy and cancer missense mutations
Source: Mol Syst Biol. 2014 May 6;10(5):727. doi: 10.1002/msb.20145092 (PMC4188041; doi:10.1002/msb.20145092)
Supplement: Supplementary file 16 — Supplementary Table S4 [file MSB-10-5-727-s16.pdf]

	Rate 1 [s-1]	Rate 2 [s-1]	Rate 4 [s-1]	Rate 6 [s-1]	Rate 7b [s-1]
WT	3.00E-05	9.300E-03	0.30	7.76	5.22
G12V	2.00E-05	1.500E-03	0.17	0.00	28.00
K5N	4.00E-05	9.400E-03	0.37	8.59	3.80
V14I	8.90E-04	1.000E-02	5.90	9.01	32.27
Q22E	7.80E-04	1.030E-02	7.00	0.25	33.46
Q22R	4.00E-05	6.400E-03	0.67	0.33	12.10
P34L	8.00E-05	9.200E-03	0.47	0.00	654.87
P34R	6.00E-05	8.900E-03	0.20	0.00	567.08
T58I	1.30E-04	5.400E-03	0.50	6.84	32.98
G60R	4.00E-05	7.000E-04	0.00	0.00	270.49
E153V	5.00E-05	4.400E-03	0.67	6.76	14.00
F156L	1.90E-03	7.900E-03	9.93	0.25	118.64

Supplementary Table S4. Rate constants of the network model to simulate Ras WT and missense mutations. Rate constants are based on Gremer *et al*, 2011 (Human Mutation 32, 33-43) See also Supplementary table S5 and Methods.
