# Supplementary material for: Structure‐energy‐based predictions and network modelling of RASopathy and cancer missense mutations
Source: Mol Syst Biol. 2014 May 6;10(5):727. doi: 10.1002/msb.20145092 (PMC4188041; doi:10.1002/msb.20145092)
Supplement: Supplementary file 17 — Supplementary Table S5 [file MSB-10-5-727-s17.pdf]

	k_{GDP} exch. intr. [s-1]	k_{GTP} hydr.intr. [s-1]	k_{obs} GEF [s-1]	k_{obs} GAP [s-1]	Kd (RAS-RAF1) [μM]
WT	3.00E-05	9.30E-03	0.09	9.3	0.22
G12V	2.00E-05	1.50E-03	0.05	0.0	1.18
K5N	4.00E-05	9.40E-03	0.11	10.3	0.16
V14I	8.90E-04	1.00E-02	1.77	10.8	1.36
Q22E	7.80E-04	1.03E-02	2.1	0.3	1.41
Q22R	4.00E-05	6.40E-03	0.2	0.4	0.51
P34L	8.00E-05	9.20E-03	0.14	0.0	27.6
P34R	6.00E-05	8.90E-03	0.06	0.0	23.9
T58I	1.30E-04	5.40E-03	0.15	8.2	1.39
G60R	4.00E-05	7.00E-04	0.0	0.0	11.4
E153V	5.00E-05	4.40E-03	0.2	8.1	0.59
F156L	1.90E-03	7.90E-03	2.98	0.3	5.0

Supplementary Table S5. Experimental rate constants for Ras RASopathy and cancer mutations. Experimental rate constants based on Gremer et al. 2011, Human Mutation 32, 33-43. k_{GDP} exch. intr. corresponds to the intrinsic GDP dissociation. k_{GTP} hydr.intr. corresponds to the intrinsic GTP hydrolysis. k_{obs} GEF corresponds to the GEF catalyzed GDP dissociation. k_{obs} GAP corresponds to the GAP catalyzed GTP hydrolysis. Kd (RAS-RAF1) corresponds to the affinity between Ras and effector protein.
