# Supplementary material for: Structure‐energy‐based predictions and network modelling of RASopathy and cancer missense mutations
Source: Mol Syst Biol. 2014 May 6;10(5):727. doi: 10.1002/msb.20145092 (PMC4188041; doi:10.1002/msb.20145092)
Supplement: Supplementary file 18 — Supplementary Table S6 [file MSB-10-5-727-s18.pdf]

Species	Initial concentration [mol l-1]
RasD	6.1e-05
RasT	0
GEF	2.56e-5
RasD-GEF	0
GAP	3.93e-05
RasT_GAP	0
EFF	3.27e-05
RasT_EFF	0

Supplementary Table S6. Initial concentrations of the network model to simulate Ras WT and missense mutations. See also Methods.
